# Highly efficient genome editing for single-base substitutions using optimized ssODNs with Cas9-RNPs

**DOI:** 10.1038/s41598-019-41121-4

**Published:** 2019-03-18

**Authors:** Sachiko Okamoto, Yasunori Amaishi, Izumi Maki, Tatsuji Enoki, Junichi Mineno

**Affiliations:** CDM Center, Takara Bio Inc. Nojihigashi 7-4-38, Kusatsu, Shiga, 525-0058 Japan

## Abstract

Target-specific genome editing using engineered nucleases has become widespread in various fields. Long gene knock-in and single-base substitutions can be performed by homologous recombination (HR), but the efficiency is usually very low. To improve the efficiency of knock-in with single-stranded oligo DNA nucleotides (ssODNs), we have investigated optimal design of ssODNs in terms of the blocking mutation, orientation, size, and length of homology arms to explore the optimal parameters of ssODN design using reporter systems for the detection of single-base substitutions. We have also investigated the difference in knock-in efficiency among the delivery forms and methods of Cas9 and sgRNA. The knock-in efficiencies for optimized ssODNs were much higher than those for ssODNs with no blocking mutation. We have also demonstrated that Cas9 protein/sgRNA ribonucleoprotein complexes (Cas9-RNPs) can dramatically reduce the re-cutting of the edited sites.

## Introduction

CRISPR-Cas9 genome editing holds promise in a wide variety of fields^1–4^. Single-base substitutions using ssODNs can be used to introduce and/or correct disease-associate mutations in order to generate human disease models for drug discovery and the elucidation of diseases^5–7^. After a targeted double-stranded break (DSB) by the CRISPR-Cas9 system, the genome is typically repaired by non-homologous end-joining (NHEJ), inducing gene knock-out by insertions and deletions (Indels). Gene knock-in and the introduction of precise mutations can be achieved by homology-directed repair (HDR) using a donor DNA template, but the efficiency is usually very low, which hampers the development of this technique for clinical application. In the case of long DNA knock-in using long donor DNA templates that include drug resistance genes, the correctly edited cells can be concentrated by drug selection even when the HDR efficiency is low. However, when ssODNs are used as donor templates, the correctly edited cells cannot be concentrated, so the introduction efficiency of single-base substitutions tends to be very low, and obtaining edited clones with the desired mutations is difficult^7,8^.

Several approaches to improving the HDR efficiency have been explored. Chemical reagents to arrest the cell cycle were examined, and nocodazole treatment was reported to improve the HDR efficiency because HDR occurs only in the late S and G2 phases, whereas NHEJ predominates throughout the cell cycle^9^. In addition, small molecules that inhibit NHEJ or enhance HDR, such as brefeldin A^10^, KU-0060648^11^, L-755,507^10^, NU7441^11^, RS-1^12,13^, and SCR7^14,15^ were reported to improve HDR efficiency. However, since the other unexpected influences caused by those small molecules are still unknown, genome editing without any additional factors is more feasible. Other approaches to improving HDR efficiency have been reported, including the stabilization of ssODNs^16,17^ and sgRNAs^18^ by chemical modification and the optimization of ssODN design. The length and orientation of mutated ssODNs were optimized for altering an sgRNA target sequence to a non-target sequence, and 70 nt or asymmetric ssODNs complementary to the sgRNA strand were reported to improve the editing efficiency^6,19,20^. In single-base substitution by CRISPR/Cas9 systems, re-cutting of the edited sites was reported, and a silent mutation to block the re-cutting increased the HDR accuracy^17,20,21^. However, no previous report has evaluated the effects of both blocking mutations and other aspects of ssODN design on knock-in efficiency. In this study, using our novel reporter system, which can detect both the gene knock-out efficiency of Indels and single-base substitutions by accurate genome editing, we have evaluated the effect of the blocking mutation, orientation, size, and length of the homology arms of ssODNs on the efficiency of single-base substitutions.

Recently, the delivery of Cas9-RNPs was reported to show more efficient on-target cleavage and reduce off-target cleavage compared to the results of plasmid transfection^22–24^. Furthermore, genome editing using Cas9-RNPs can solve problems such as the random integration and insertion of plasmids into the genome at on-/off-target sites and the severe cytotoxicity caused by the introduction of nucleic acids. In addition to those advantages, we have evaluated the feasibility of using Cas9-RNPs for single-base substitutions.

For successful genome editing in induced pluripotent stem cells (iPSCs), in addition to improving the editing efficiency, increasing the single-cell cloning efficiency and maintaining iPSCs in an undifferentiated state throughout the whole process are essential^25–28^. In this study, we have evaluated the feasibility of the feeder-free culture system for hiPSCs, the Cellartis^®^ DEF-CS^TM^ culture system in the single-cell cloning of genome-edited human iPSCs (hiPSCs) to obtain undifferentiated and correctly edited clones.

## Results

### The evaluation system for the detection of accurate single-base substitutions

**Figure 1:**
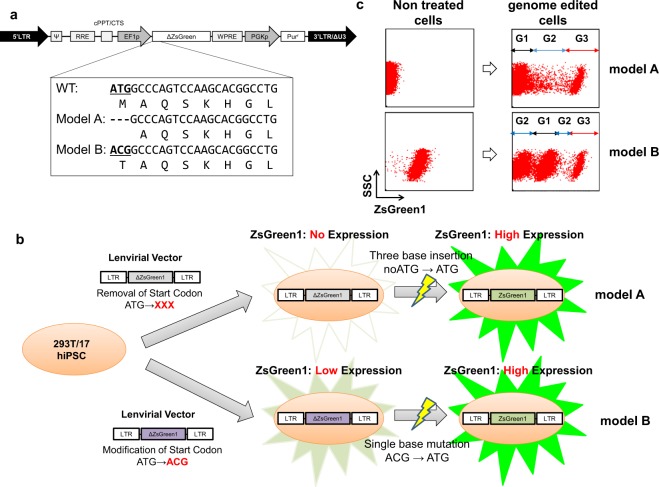
Evaluation systems for single-base substitutions by genome editing. (**a**) Lentiviral vectors expressing ZsGreen1 were constructed. The start codon “ATG” was removed in model A and altered to “ACG” in model B. (**b**) Schematic diagram of the evaluation systems for model A and model B. (**c**) Representative flow cytometry analysis data of untreated and genome-edited model clones, 293T-A1 (upper) and 293T-B1 (lower). The cells in G1 are unmodified cells and cells with Indels that did not affect the ZsGreen1 expression, the cells in G2 are cells with Indels that did affect the expression, and the cells in G3 are correctly modified cells.

**Figure 2:**
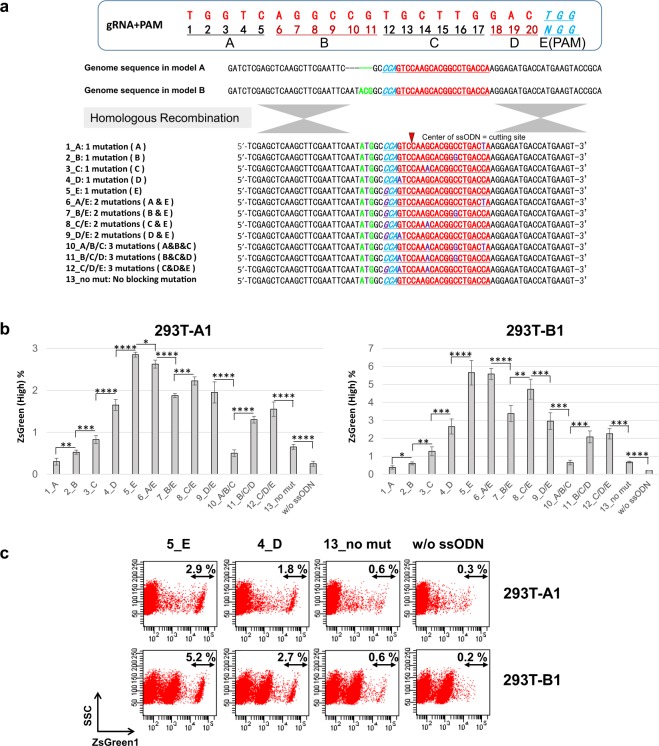
The effect of blocking mutations in ssODNs on knock-in efficiencies. (**a**) The sequences of the gRNA and genomic DNA in model A and model B and the list of ssODNs and their sequences used as donor templates. (**b**) The knock-in efficiencies assessed by the percentages of cells with high ZsGreen1 expression in the 293T-A1 and 293T-B1 model clones transfected with plasmids expressing Cas9 and sgRNA and with 50 pmol of ssODNs. The data shown are the means ± SD of four different transfected samples from two independent experiments, and evaluated by Student’s t-test. *P < 0.05, **P < 0.01, ***P < 0.005, and ****P < 0.001. (**c**) Representative flow cytometry analysis of the transfected 293T-A1 and 293T-B1 cells in (**b**).

To evaluate the knock-in efficiency obtained with ssODNs using the CRISPR/Cas9 system, we have established evaluation systems using ZsGreen1 as a target gene for the simultaneous detection of accurate single-base substitutions and Indel mutations in both 293T cells and hiPSCs using a flow cytometer. We constructed lentiviral vector plasmids for models A and B. The plasmid for model A contains the ZsGreen1 gene lacking the start codon “ATG”, which can introduce ZsGreen1 gene without protein expression, and the plasmid for model B contains the ZsGreen1 gene with very weak expression, caused by altering the start codon “ATG” to “ACG”^29,30^ to reduce the fluorescein protein expression level (Fig. 1a,b). Then, 293T/17 cells and 253G1 hiPSCs^31^ were transduced with each lentivirus, and after puromycin selection, model clones containing one or two proviral copy numbers were established. We selected the 293T-A1 and 293T-A2 model clones (containing 1 and 2 copies, respectively) for model A and the 293T-B1 and 293T-B2 model clones (containing 1 and 2 copies, respectively) and the hiPS-B2 model clone (containing 2 copies) for model B. We have confirmed the sequence of inserted modified-ZsGreen1 genes have no mutation (data not shown). Using these novel systems, we introduced plasmids expressing Cas9 and sgRNA, together with ssODN containing the normal start codon “ATG” (ssODN-5_E in Fig. 2a) as a recombinant template into the model clones. We could detect the accurate single-base correction in model A and both the accurate single-base correction and the Indel mutations simultaneously in model B using a flow cytometer (Fig. 1c). To validate this novel evaluation system (See Supplementary Fig. S1), we performed a knock-in experiment using the 293T-B1 model clone with ssODN-5_E, as shown in Fig. 2a, and sorted the cells with high ZsGreen1 expression in gate D, the cells without expression in gate A, and the cells with slightly increased expression level in gate C (Fig. S1a), and confirmed that all the clones in gate A (purity of 97.5%) had frameshift mutations resulting from the Indel mutation (Fig. S1b). As the purity of the sorted cells in gate C was not high (purity of 76.7%), and 7 out of 16 clones were identified as unedited clones contaminated from the gate B. However, rest of the clones (9 out of 16 clones) had Indel mutations with newly created in-frame start codon “ATG” which may slightly increase the ZsGreen1 expression (Fig. S1c). Using the enriched cells in gate D (purity of 94.1%), we analysed the sequence of the target site of the genome. Almost 94% of the clones (17 out of 18 clones) had the desired sequence with the corrected start codon “ATG”, but 4 out of 17 clones also had Indels upstream of the start codon, which did not affect the ZsGreen1 expression (Fig. S1d). Using this novel reporter model system, we could roughly detect both knock-in and knock-out events using a flow cytometer without performing single cell cloning and sequence analysis of each genome-edited clone, which is absolutely essential for real-life applications.

### The optimal design of ssODNs for single-base substitutions by HR

To improve the knock-in efficiencies obtained using ssODNs as donor templates, we have optimized the design of ssODNs. The ssODNs were designed to restore the start codon “ATG” with one to three additional base mutation(s) at various positions to block the re-cutting of the correctly edited genome. The gRNA sequence used to target ZsGreen1 was divided into 5 regions, A, B, C, D, and E (=PAM), and all the blocking mutations were designed to be silent mutations that would not influence the expression of ZsGreen1, which is analysed by the model system (Fig. 2a). The plasmids expressing Cas9 and sgRNA were co-introduced with each ssODN into the 293T-A1 and B1 model clones, and the knock-in efficiencies were compared using a flow cytometer. The ssODNs with a mutation at PAM showed the highest knock-in efficiency, and an inverse correlation was observed between the distance from PAM to the mutation site in the ssODN and the knock-in efficiencies in both 293T models. Furthermore ssODN-10_A/B/C with multiple PAM distal mutations in seed region showed much lower knock-in efficiencies (Fig. 2b,c). These results indicate that the re-cutting of the corrected gene is the main cause of very low efficiencies, and the presence of a block mutation at PAM proximal sites not at PAM distal sites to prevent re-cutting is essential for efficient knock-in.

**Figure 3:**
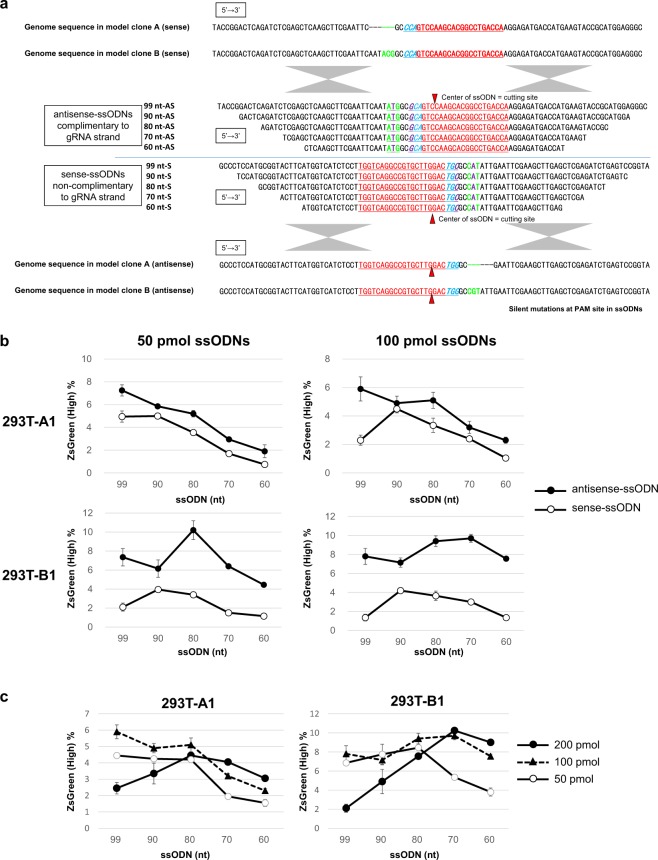
The optimal strand orientation and length of ssODNs. (**a**) The sequences of the genomic DNA in model A and model B and the list of ssODN sequences used as donor templates with a silent mutation at the PAM sequence. (**b**) The knock-in efficiencies assessed by the percentages of cells with high ZsGreen1 expression in the 293T-A1 and 293T-B1 model clones transfected with plasmids expressing Cas9 and sgRNA and with 50 or 100 pmol of antisense and sense ssODNs. The data shown are the means ± SD of two independent transfected samples. The transfection experiments with 50 pmol and 100 pmol of ssODNs were performed independently. (**c**) The knock-in efficiencies assessed by the percentages of cells with high ZsGreen1 expression in 293T-A1 and 293T-B1 model clones transfected with plasmids expressing Cas9 and sgRNA and with 50, 100, or 200 pmol of antisense ssODNs. The data shown are the means ± SD of two independent transfected samples.

**Figure 4:**
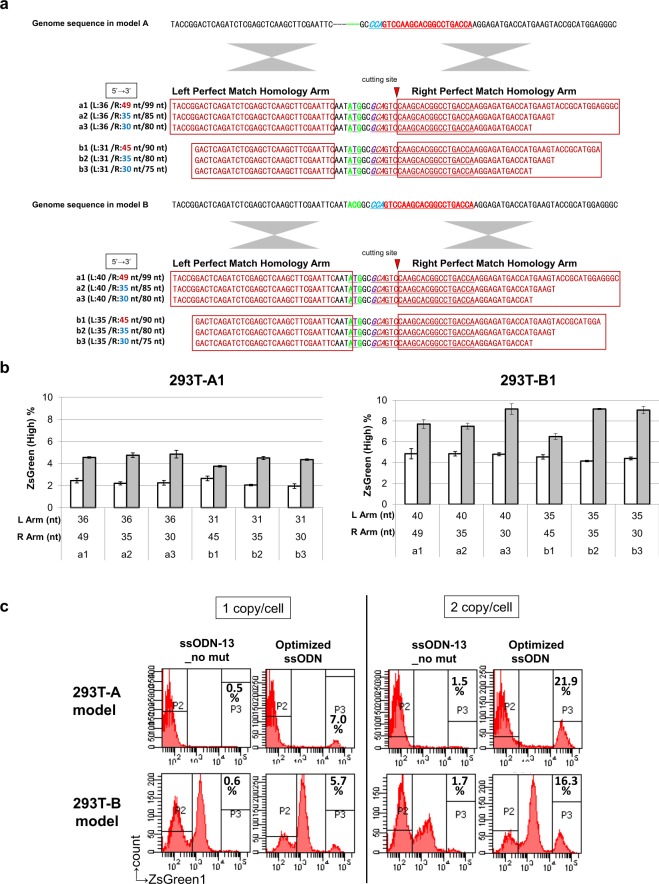
The optimal length of the homology arms in ssODNs. (**a**) The sequences of the genomic DNA in model A and model B and the list of ssODN sequences used as donor templates with a silent mutation at the PAM sequence. The lengths of the left homology arm (L), the right homology arm (R), and the total ssODNs are shown. (**b**) The knock-in efficiencies assessed by the percentages of cells with high ZsGreen1 expression in 293T-A1 and 293T-B1 model clones transfected with plasmids expressing Cas9 and sgRNA and with 25 pmol (white bar) or 50 pmol (grey bar) of ssODNs. The data shown are the means ± SD of duplicated experiments, representative of two independent experiments. The same experiments using the selected ssODNs and different model clones were repeated several times with similar results. (**c**) The unoptimized ssODN (ssODN_13-no mut in Fig. 2a) and the optimized ssODN (7_Optimized ssODN) shown in Fig. 5a were co-introduced with Cas9 and sgRNA expression plasmids into the 293T-A1, 293T-A2, 293T-B1, and 293T-B2 models. Representative flow cytometry analysis results for the transfected model clones and the percentages of P3 gates, which indicate the knock-in efficiency, are shown.

**Figure 5:**
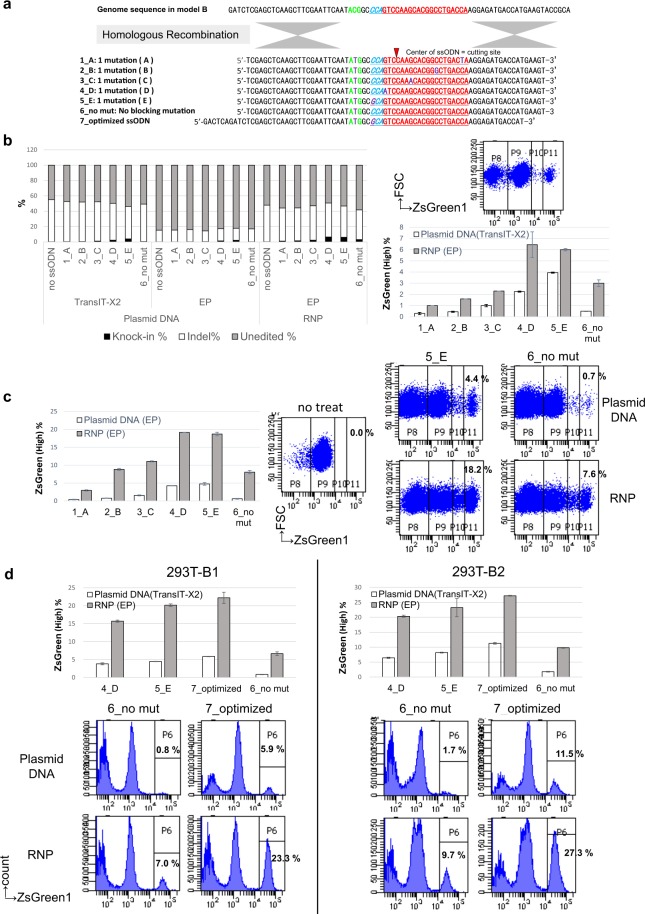
The optimal delivery methods for Cas9 protein and sgRNA for single-base substitution. (**a**) The sequences of the genomic DNA in model B and the list of ssODN sequences used as donor templates with a silent mutation (except for 6_no mut, which has no blocking mutation and is the same ssODN as 13_no mut in Fig. 2a). (**b**) Cas9 protein and sgRNA were introduced into 293T-B1 model clones as plasmid DNAs or Cas9-RNPs with or without 50 pmol of ssODNs using the TransIT-X2 transfection reagent or by electroporation (EP). Representative flow cytometry analysis data is shown upper right to clarify the gates. The left graph shows the knock-in % (gate P11), Indel % (the sum of gate P8 and P10), and unedited % (gate P9). The data shown are the mean values of duplicated samples, and two independent experiments were repeated with similar results. The lower right graph shows the only knock-in % of the 293T-B1 model clones. The data shown are the means ± SD of duplicated samples, and two independent experiments were performed with similar results. (**c**) The graph shows the knock-in % of the hiPS-B2 model clones with Cas9 protein and sgRNA introduced as plasmid DNAs or Cas9-RNPs with 50 pmol of ssODNs by EP. The data shown are the means ± SD of duplicated samples, and two independent experiments were performed with similar results. Representative flow cytometry analysis data of the model clones and the percentages of P11 gates, which indicate the knock-in %, are shown. (**d**) The graph shows the knock-in % of the 293T-B1 and 293T-B2 model clones with Cas9 protein and sgRNA introduced as plasmid DNAs or Cas9-RNPs with 100 pmol of ssODNs using the TransIT-X2 transfection reagent or by EP. The data shown are the means ± SD of duplicated samples, and two independent experiments were performed with similar results. Representative flow cytometry analysis data of both model clones and the percentages of P6 gates, which indicate the knock-in efficiency, are shown.

We also designed both sense and antisense ssODNs of different lengths (60 to 99 nt). The sense ssODNs were the same strand and the antisense ssODNs the complementary strand to the gRNA, and all ssODNs contained a blocking mutation at the PAM site based on the sequence of ssODN-5_E shown in Fig. 2a (Fig. 3a). Then, 50 or 100 pmol of ssODNs was introduced with plasmids expressing Cas9 and sgRNA into 293T-A1 and B1 to compare the knock-in efficiencies. All the model clones transfected with antisense ssODNs showed higher knock-in efficiencies than the clones transfected with sense ssODNs at all lengths. In addition, longer ssODNs with longer homology arms showed higher HR efficiencies, but the knock-in efficiency reached a plateau at 80 or 90 nt in both model clones (Fig. 3b). For further evaluation, we selected antisense ssODNs and performed knock-in experiments in the 293T model clones using different doses of ssODNs (50, 100, and 200 pmol). The cells treated with 200 pmol of ssODNs showed the larger proportion of round-cell morphology than other conditions at 2 days after transfection, indicating the highest amount of ssODNs caused cytotoxicity and resulted in low knock-in efficiency, and in addition longer ssODNs at the highest amount showed a significant reduction in efficiency (Fig. 3c). These results indicate that the balance between the HR efficiency and the cytotoxicity of ssODNs is important for efficient single-base substitution by genome editing. To determine the optimal length of the perfectly matched homology arm in ssODNs, we shortened only the right homology arm from 49 or 45 nt to 35 or 30 nt in the ssODN-99 nt-AS: a1 in Fig. 4a and the ssODN-90 nt-AS: b1 in Fig. 4a (Figs 3a and 4a). When we introduced 50 pmol of ssODNs with Cas9 and sgRNA plasmids, at the dose at which longer ssODNs caused cytotoxicity, we could observe a slight decrease in knock-in efficiency with longer ssODNs; therefore, shortening the right homology arm slightly increased the knock-in efficiency. On the other hand, when a lower amount (25 pmol) of ssODNs was used, no significant difference was observed among the ssODNs (Fig. 4b). Based on these results, we have concluded that the optimal ssODN for introducing a single-base substitution has a mutation at the PAM sequence or, if not possible, at the 5′ neighbouring base of PAM; is complementary to the gRNA strand; and has a total length of approximately 75–85 nt with 30 to 35 nt perfectly matched homology arms on both sides. The optimized ssODN in Fig. 5a shows the perfect balance between HR efficiency and cytotoxicity, resulting in much higher knock-in efficiency than the ssODN-13_no mut in Fig. 2a with no blocking mutation in the 293T-A1, A2, B1, and B2 model clones (Fig. 4c).

### Superiority of Cas9-RNPs for single-base substitutions

Next, we investigated the delivery methods of Cas9 protein and sgRNA and compared the genome editing efficiencies using plasmids and RNPs. Using 293T-B1, we introduced the plasmids or RNPs for Cas9 and sgRNA together with ssODNs (Fig. 5a) and compared the knock-in and Indel efficiencies. In the 293T-B1 clone, with 1 vector copy, the total editing efficiencies (both knock-in and Indel) were almost constant for the same delivery method regardless of ssODN design, and the introduction of plasmids expressing Cas9 and sgRNA together with ssODN using a polymeric delivery reagent, TransIT-X2 showed higher editing efficiency than delivery by electroporation (Fig. 5b). To confirm the results from 293T model system are applicable to other cell model, we have introduced the plasmids or RNPs for Cas9 and sgRNA together with ssODNs by electroporation in hiPS-B2 clone. In both 293T-B1 and hiPS-B2, RNP delivery showed higher knock-in efficiency than plasmid delivery regardless of the ssODNs. In both model cells, the knock-in efficiency was the highest when using ssODNs with mutation at the PAM site (ssODN-5_E) or at the 5′ neighbouring base of PAM (ssODN-4_D) as the donor DNA, regardless of the delivery method of Cas9 and sgRNA. However, the reduction of knock-in efficiency by RNPs with ssODNs having no blocking mutation was much smaller than that of plasmids, indicating that RNPs could dramatically reduce the re-cutting of the edited sites (Fig. 5b,c). Next, we optimized the ratio of Cas9 protein, sgRNA, and ssODNs for efficient single-base substitutions. Using ssODN-4_D, we found that 1 µg of Cas9 protein, 0.4 µg of sgRNA, and 100 pmol of ssODN showed the highest knock-in efficiency (data not shown). Then, using this condition, we compared the knock-in efficiencies among ssODNs, including the optimized ssODN, by introducing Cas9 protein and sgRNA using plasmids or RNPs into the 293T-B1 and B2 model clones. In both model clones and by both delivery methods, the optimized ssODN (7_optimized ssODN) showed the highest knock-in efficiency among all ssODNs tested. The delivery of Cas9 RNP with the optimized ssODN showed the highest knock-in efficiency (Fig. 5d). We have also designed several gRNAs to induce DSBs at various distance from the base-substitution site (start codon ATG) in ZsGreen1, and ssODNs for each gRNA with or without blocking mutation at PAM proximal site containing 30–35 nt perfect homology arms at both ends (See Supplementary Tables S1 and S2). Having the longer distance from the DSB sites and base-substitution site (24, 52, 97 nt), the total length of newly designed ssODNs were longer than our optimized ssODN size, and the knock-in efficiencies by electroporation of RNP and ssODNs were compared in 293T-B1 model. As each sgRNA has different cutting activity, we have also calculated the knock-in efficiency in all edited cells (both knock-in and Indel) to normalize the cutting efficiency (See Supplementary Table S3). The knock-in efficiencies were slightly increased in the sample edited with ssODNs containing a blocking mutation for sgRNA-A and B, but decreased dramatically when we use sgRNA-A, B, and C which induce DSBs at longer distal sites from the target base for substitution. Therefore, the distance between DSB site and target base is another critical point for single-base substitutions, as previously reported^17,21^. We have also constructed other reporter systems utilizing acGFP1 eliminating start codon “ATG” (See Supplementary Fig. S2). As the gRNA used for this model induce the DSBs distal from the target base, the length of G_optimized ssODN is longer than our criteria (Fig. S2b). As shown in Fig. S2c, in both 293T-C1 and C2 models, the knock-in efficiencies were higher when using G_optimized ssODN with blocking mutation as the donor DNA, regardless of the delivery method of Cas9 and sgRNA, although the knock-in efficiency was really low by plasmids transfection. However, the reduction of knock-in efficiency by RNPs with ssODNs having no blocking mutation was also relatively small in AcGFP1 models (Fig. S2b,c).

### The optimal system for the single-cell cloning step to promote expansion of the hiPSC clones without loss of pluripotency

**Figure 6:**
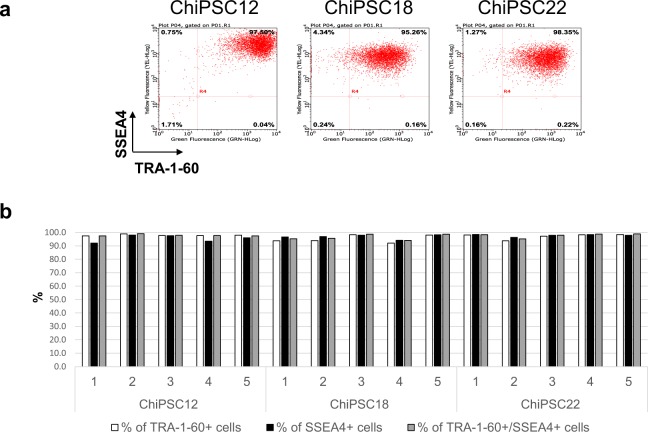
Pluripotency marker expression in the hiPSC clones. Three different hiPS cell lines (ChiPSC12, 18, and 22) were seeded in 96-well plates at 1 cell/well, and the clones obtained were stained with anti-TRA-1-60 and SSEA4 antibodies. (**a**) Representative flow cytometry analysis of the pluripotency marker expression for each iPS cell line. (**b**) The percentages of TRA-1-60-positive, SSEA-4-positive, and double-positive cells in 5 distinct clones for each iPS cell line.

To evaluate a feeder-free hiPSC culture system in the single-cell cloning step, three different hiPS cell lines (ChiPSC12, 18, and 22) were cultured in the Cellartis^®^ DEF-CS^TM^ culture system, and were seeded at 1 cell/well in 96-well plates. The culture system showed the high cloning efficiency (about 30% of cloning efficiencies with all three hiPS cell lines), and we could expand all the clones we have tested. Then we examined the expression of pluripotency markers SSEA-4 and TRA-1–60 in the expanded clones from all hiPS cell lines, and all the clones maintained the expression of the pluripotency markers (Fig. 6a,b).

## Discussion

We have investigated methods to improve the efficiency of single-base substitutions by CRISPR/Cas9 genome editing with minimum effect on the cells apart from the introduction of components essential for genome editing. Using plasmids expressing Cas9 and sgRNA, we have demonstrated that blocking mutations in ssODNs to prevent the re-cutting of the edited site can greatly improve the knock-in efficiency, and ssODNs with a mutation at PAM showed the highest knock-in efficiency using reporter systems. An inverse correlation was observed between the distance from PAM to the mutation site in ssODNs and the knock-in efficiency. These results are consistent with previous reports showing that the bases of the target sequences that are distant from the PAM sequence are less important for Cas9 specificity and the PAM proximal mutation in ssODN is important for efficient knock-in^17,20,21,32–36^. The ssODNs we used, shown in Fig. 2a, have various lengths of perfectly matched homology arms. Although the length of the perfectly matched homology arms may influence the HR efficiency, the knock-in efficiency results using ssODNs with 2 or 3 blocking mutations did not correlate with the length of the homology arms, indicating that the position of the blocking mutation greatly influenced the knock-in efficiency and that the presence of PAM proximal blocking mutations in ssODNs to prevent re-cutting is essential for efficient knock-in. Our data also indicated that longer ssODNs with longer homology arms increased the HR efficiency to some extent, but longer ssODNs also showed severe cytotoxicity that decreased the knock-in efficiency, so the length of the ssODNs should be optimized to balance the HR efficiency and the cytotoxicity. Using our novel models, we have optimized the design of the ssODNs as follows: the strand is complementary to the gRNA strand, the total length is approximately 75–85 nt, the length of each perfect match homology arm is 30–35 nt, and a blocking mutation is present at or near the PAM sequence (Figs 2, 3, and 4). The optimized ssODNs showed much higher knock-in efficiencies than the ssODNs with no blocking mutation when introduced with plasmids expressing Cas9 and sgRNA (Fig. 4c). In addition to the optimized designs listed above, it is also important to select gRNAs to induce DSBs at proximal to the target base for substitution as previously reported^17,21^ (Table S3).

We have also demonstrated that RNPs can improve the knock-in efficiencies in both our novel 293T and hiPSC models. Since the total editing efficiencies (both knock-in and Indel) in the 293T-B1 model transfected with Cas9 and sgRNA plasmids using TransIT-X2 were similar to those in the cells into which Cas9-RNPs were introduced, plasmid and RNP delivery made little difference in the specific cutting activity of Cas9 and sgRNA. Accordingly, we can conclude that the timing of the presence of the ssODNs at the DSB sites may be very important for efficient HR. In the 293T model, the knock-out efficiency was higher in cells modified with Cas9-RNPs than in cells into which plasmids were delivered by electroporation. This difference may occur because proteins are less cytotoxic than nucleotides such as plasmid DNA, and the delivery of Cas9 and sgRNA plasmids therefore induced higher cytotoxicity^23^, resulting in lower total editing efficiency (Fig. 5b). The knock-in efficiency was the highest when using ssODNs with the blocking mutation at the PAM site or at the 5′ neighbouring base of PAM as donor DNAs, which was similar to the results using plasmids. However, the reduction in knock-in efficiency with ssODNs having no blocking mutation was much smaller when using RNPs than when using plasmids, indicating an additional advantage of RNPs, whose short lifetime in the cell can dramatically reduce the re-cutting of the edited sites and thereby improve the knock-in efficiency (Figs 5b–d, and S2c).

Human disease models are indispensable for drug screening and disease investigation, however, obtaining sufficient numbers of disease cells from patients is very difficult. The discovery of hiPSCs and the development of differentiation technology to obtain matured cells from hiPSCs enabled researchers to prepare sufficient numbers of diseased model cells by differentiation from the patient’s hiPSCs^26–28^. However, obstacles sometimes occur in obtaining cells derived from patients, and large variations in disease-related and disease-unrelated SNPs exist among patients. Therefore, the use of uniform cells with specific disease-related SNPs is more feasible. The application of genome editing technology to hiPSCs has enabled the creation of disease model cells by differentiation from genome-edited hiPSCs created using disease-related SNPs. To establish disease model hiPSCs, in addition to improvements in genome editing efficiency, an optimal system for the single-cell cloning step is crucial to promote clone expansion and to maintain pluripotency throughout the process of the genome editing of hiPSCs. We have confirmed the utility of feeder-free hiPSC culture system, the Cellartis^®^ DEF-CS^TM^ culture system in the single-cell cloning and expansion steps of hiPSCs. Moreover, all the expanded clones in the Cellartis^®^ DEF-CS^TM^ culture system maintained the expression of pluripotency markers, showing the feasibility of the Cellartis^®^ DEF-CS^TM^ culture system for obtaining genome-edited hiPSC clones (Fig. 6).

In summary, using our reporter systems, we were able to demonstrate a highly efficient system for obtaining genome-edited cells with single-base substitutions using Cas9 RNPs delivered with optimized ssODNs as donor DNAs, without using additional components that might cause unwanted effects on the cells. Our findings will provide hints of the optimal ssODNs’ designs and strategy for efficient single-base substitutions.

## Methods

### Cell lines

The 293T/17 (HEK 293T/17) (ATCC^®^ CRL-11268™) cell line was cultured in DMEM (Sigma-Aldrich) supplemented with 10% FBS, penicillin (100 U/ml), and streptomycin (100 μg/ml). The human iPS cell lines 253G1, Cellartis^®^ human iPS cell line 12 (ChiPSC12), Cellartis^®^ human iPS cell line 18 (ChiPSC18), and Cellartis^®^ human iPS cell line 22 (ChiPSC22) (TAKARA BIO), were cultured using the Cellartis^®^ DEF-CS™ 500 Culture System (TAKARA BIO).

### Construction of ZsGreen 1 expression lentiviral vectors

The plasmid vector pLVSIN-EF1-IRES-ZsGreen1 (TAKARA BIO) was digested by BamHI and NcoI, and the excised IRES-ZsGreen1 gene was inserted into the BamHI and NcoI site of pLVSIN-EF1-acGFP1-N1 (TAKARA BIO) to construct pLVSIN-EF1-IRES-ZsGreen1-puro. In addition, the start codon “ATG” of ZsGreen1 was removed (for model A) or replaced with “ACG” (for model B) using the PrimeSTAR^®^ Mutagenesis Basal Kit (TAKARA BIO) to construct the plasmids pLVSIN-EF1-IRES-ΔZsGreen1-noATG-puro and pLVSIN-EF1-IRES-ACG-ΔZsGreen1-puro, which were then used as the templates for PCR using specific primers (forward primer for model A: 5′-ATAGAATTCGCCCAGTCCAAGCACGGCCT-3′; forward primer for model B: 5′-GCGGAATTCAATACGGCCCAGTCCAAGCA-3′; reverse primer: 5′-GGAGCAACATAGTTAAGAATACCAGTC-3′). The amplified DNA fragments were digested with EcoRI and MluI and cloned into the same sites of pLVSIN-EF1-IRES-ΔZsGreen1-noATG-puro and pLVSIN-EF1-IRES-ACG-ΔZsGreen1-puro to obtain pLVSIN-ΔZsGreen1-noATG-puro-N (model A) and pLVSIN-ΔZsGreen1-ACG-puro-N (model B).

### Lentiviral vector production and the establishment of the model cell clones

The lentiviruses were produced using Lentiviral High Titer Packaging Mix (TAKARA BIO) in 293T/17 cells and used to transduce 293T/17 cells and 253G1 cells at several dilution folds. The transduced cell populations were cultured in medium containing puromycin (TAKARA BIO) at concentrations of 2 μg/mL and 0.5 μg/mL, respectively. Then, several clones were obtained by limiting dilution in 96-well plates, and the 293T/17 clones with 1 and 2 viral genome copies and the 253G1 clones with 2 copies were selected for the model system.

### Proviral copy number analysis in lentiviral transduced model clones

qPCR was performed with a set of specific primers to detect either the lentiviral vector sequence in the Lenti-X™ Provirus Quantitation Kit (Clontech) or the human interferon gamma (hIFNγ) gene in the Provirus Copy Number Detection Primer Set, Human (for Real Time PCR) (TAKARA BIO) using SYBR^®^ Premix Ex Taq™ II (Tli RNaseH Plus) (TAKARA BIO) to determine the ratio between the copy numbers of the provirus gene and hIFNγ gene. The provirus gene and the hIFNγ gene were quantified in two separate reactions using the genomic DNA extracted from the model clones.

### sgRNA plasmids and sgRNA production

The plasmids expressing sgRNAs were constructed using the pGuide-it-sgRNA1 Vector System (TAKARA BIO), and the sgRNAs were produced using the Guide-it™ sgRNA *In Vitro* Transcription Kit (TAKARA BIO) according to the manufacturer’s protocols.

### Transfection

To perform genome editing, 1.75 × 10^5^ cells of the 293T/17 model clones were transfected with 0.25 µg of SpCas9 plasmid (ToolGene), 0.2 µg of the sgRNA plasmid, and 25–200 pmol of the ssODN in 24-well plates using 1.5 µL of TransIT-X2 (Mirus) according to the manufacturer’s protocol. For RNP transfection, 1.0 µg of Guide-it™ Recombinant Cas9 (Electroporation-Ready) (TAKARA BIO) and 0.4 µg of sgRNA were incubated at 37 °C for 5 min, mixed with 50 or 100 pmol of ssODNs, and introduced into 1 × 10^5^ cells of 293T/17 model clones and 253G1 model clones using the Neon^®^ Transfection System (Thermo Fisher Scientific) under the conditions of voltage 1100 v, width 20 ms and pulse number 2, and the transduced cells were cultured in 24-well plates.

### Flow cytometry analysis of model clones

After 6 or 7 days of transfection, the genome-edited cells were analysed using a FACSCanto™ II flow cytometer (BD Bioscience) to measure the ZsGreen1 expression and thereby evaluate the knock-in and knock-out (Indel) efficiencies.

### Single-cell cloning and pluripotency marker expression analysis

Three different hiPS cell lines ChiPSC12, ChiPSC18, and ChiPSC22 were cultured using the Cellartis^®^ DEF-CS™ 500 Culture System for three weeks and then seeded at 1 cell/well in coated 96-well plates using the Cellartis^®^ iPSC Single-Cell Cloning DEF-CS™ Culture Media Kit (TAKARA BIO). After 10 days, the number of wells containing growing cells were counted, and the cloning efficiencies were calculated, then the cells in five wells for each hiPS clone were detached and transferred into 24- or 48-well plates depending on the cell numbers, then cultured according to the manual of the Cellartis^®^ iPSC Single-Cell Cloning DEF-CS™ Culture Media Kit. After 9 days, these clones were double stained with Alexa Fluor 488-conjugated TRA-1-60 antibody (BD Bioscience) and phycoerythrin-conjugated SSEA-4 antibody (R&D Systems). The cells were analysed by flow cytometry using a Guava^®^ easyCyte flow cytometer (Merck Millipore), and the percentages of TRA-1-60-positive, SSEA-4-positive, and double-positive cells were quantified.

## Supplementary information

Supplementary Figures and Tables
