# Supplementary material for: Highly efficient genome editing for single-base substitutions using optimized ssODNs with Cas9-RNPs
Source: Sci Rep. 2019 Mar 18;9:4811. doi: 10.1038/s41598-019-41121-4 (PMC6423289; doi:10.1038/s41598-019-41121-4)
Supplement: Supplementary file 1 — Supplementary Figures and Tables [file 41598_2019_41121_MOESM1_ESM.pdf]

Highly efficient genome editing for single-base substitutions using optimized ssODNs with Cas9-RNPs.

Sachiko Okamoto[1], Yasunori Amaishi[1], Izumi Maki[1], Tatsuji Enoki[1], and Junichi Mineno[1]
[1]CDM Center, TAKARA BIO INC. NOJIHIGASHI 7-4-38, KUSATSU, SHIGA, 525-0058, JAPAN

Supplementary Fig. S1

Method

The plasmids expressing Cas9 and sgRNA were introduced into the 293T-B1 model clone with 50 pmol of ssODN-5_E shown in Fig. 2a, and the genome-edited populations were divided into 4 regions; A, B, C, and D. Then, the cells in gate A , C, and D were sorted using FACS Aria (BD Biosciences). The genomic DNA was extracted from the cells in each gate using NucleoSpin® Tissue (MACHEREY-NAGEL), and the target region was amplified by PCR using PrimeSTAR® Max DNA Polymerase (TAKARA BIO), forward primer: 5'-TTGCGTGAGCGGAAAGATGG-3', and reverse primer: 5'-GGAGCAACATAGTTAAGAATACCAGTC-3'. The amplified PCR products were used for subcloning with the Mighty TA-cloning Reagent Set for PrimeSTAR® (TAKARA BIO), and the clones were amplified and purified using NucleoSpin® Plasmid QuickPure (MACHEREY-NAGEL). The insert sequences were analysed using the PCR forward primer.

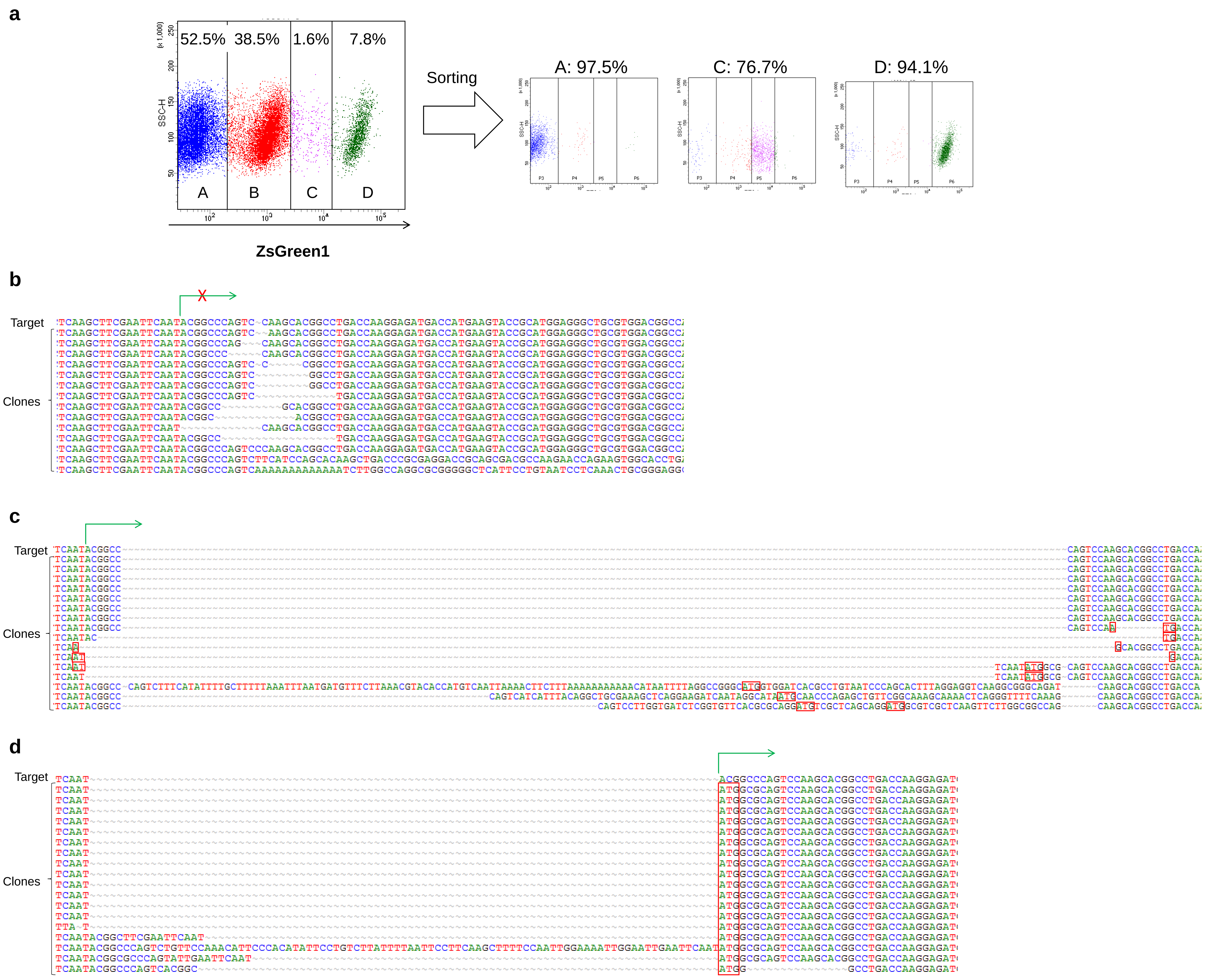

Figure. S1 Feasibility of the novel evaluation system.

After genome editing using ssODN to restore the start codon ATG in the 293T-B1 model, the cells were analysed using FACS. The cells may contain Indels with no ZsGreen1 expression in gate A, the cells may contain Indels with medium ZsGreen1 expression in gate C, and the cells with accurate substitution obtained by genome editing in gate D were sorted and collected. The sequences near the target site were analysed. (a) A diagram of the genome-edited cell analysis before FACS sorting and the sorted cells after sorting. The percentage showed in the figure indicate the purity of the sorted cells. (b) Sequence alignment of each clone from the cells in gate A. The top sequence in the alignment is the wild-type target sequence. The modified start codon "ACG" is indicated by an arrow. (c) Sequence alignment of each clone from the cells in gate C. The top sequence in the alignment is the wild-type target sequence. The modified start codon "ACG" is indicated by an arrow. The newly created in-frame "ATG" which may work as a start codon is marked with a red rectangle. (d) Sequence alignment of each clone from the cells in gate D. The top sequence in the alignment is the wild-type target sequence. The restored start codon "ATG" is marked with a red rectangle.

Highly efficient genome editing for single-base substitutions using optimized ssODNs with Cas9-RNPs.

Sachiko Okamoto[1], Yasunori Amaishi[1], Izumi Maki[1], Tatsuji Enoki[1], and Junichi Mineno[1]
[1]CDM Center, TAKARA BIO INC. NOJIHIGASHI 7-4-38, KUSATSU, SHIGA, 525-0058, JAPAN

Supplementary Fig. S2

Method
Establishment of the acGFP1 model cell clones
The plasmid vector pLVSIN-EF1-acGFP1-N1 (TAKARA BIO) was modified to remove the start codon “ATG” of AcGFP1 using the PrimeSTAR® Mutagenesis Basal Kit (TAKARA BIO) and using specific primers (forward primer: 5’- CACCGGTCGTGAGCAAGGGCGCCGAG -3’; reverse primer: 5’- TGCTCACGACCGGTGGATCCCGGGC -3’), to construct the plasmids pLVSIN-EF1-ΔacGFP1-noATG-puro. The lentiviruses were produced and the 293T/17 clones with 1 and 2 viral genome copies (293T-C1 and 293T-C2 model) were obtained as described in Methods.

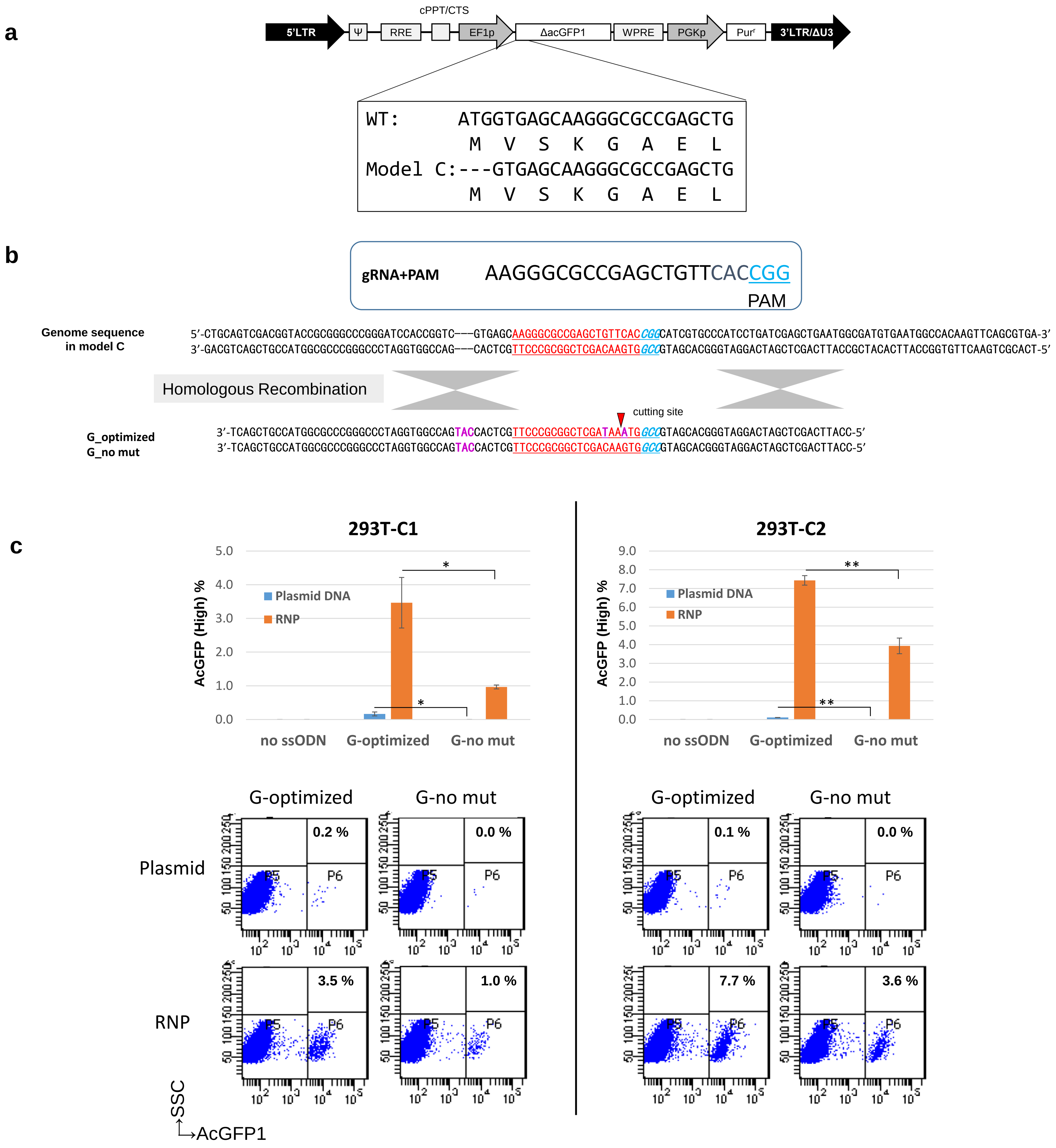

Supplemental Fig. S2
The optimal conditions for single-base substitution in AcGFP1 model.
(a) Lentiviral vector expressing AcGFP1 lacking start codon “ATG” was constructed. (b) The sequences of the gRNA and genomic DNA in model and the list of ssODNs and their sequences used as donor templates. G_optimized ssODN contains blocking mutations (silent mutation) at PAM proximal site. (c) The graph shows the knock-in % of the 293T-C1 and C2 model clones with Cas9 protein and sgRNA introduced as plasmid DNAs or Cas9-RNPs with 100 pmol of ssODNs using the TransIT-X2 transfection reagent or by EP. The data shown are the means ± SD of triplicated samples, and evaluated by Student’s t-test. *$P < 0.05$ and **$P<0.001$. Representative flow cytometry analysis data of both model clones and the percentages of P6 gates, which indicate the knock-in efficiency, are shown.

Highly efficient genome editing for single-base substitutions using optimized ssODNs with Cas9-RNPs.

Sachiko Okamoto[1], Yasunori Amaishi[1], Izumi Maki[1], Tatsuji Enoki[1], and Junichi Mineno[1]
[1]CDM Center, TAKARA BIO INC. NOJIHIGASHI 7-4-38, KUSATSU, SHIGA, 525-0058, JAPAN

Supplementary Tables (Table S1, S2, and S3)

Three different gRNAs (Table S1) and corresponding ssODNs (Table S2) were designed, and were introduced into 293T-B1 model clone as Cas9-RNPs with 100 pmol of ssODNs by EP. The knock-in (KI) % and the Indel% were measured by flow cytometry analysis, and the KI% and KI% in total edited cells (KI%/ (KI % + Indel %)) were calculated and shown in Table S3, the data shown are the means ±SD of duplicated samples, and two independent experiments were performed with similar results.

Table S1. List of gRNA sequences

	Strand	gRNA (5'→3')
original	-	TGGTCAGGCCGTGCTTGGACTGG
A	+	GTCCAAGCACGGCCTGACCAAGG
B	+	ACCATGAAGTACCGCATGGAGGG
C	+	GTGATCACCGGCGAGGGCATCGG

*: original gRNA shown in Fig.2
underline: PAM sequence

Table S2. List of ssODN sequences

name	ssODN (5'→3')
7_optimized ssODN*	GACTCAGATCTCGAGCTCAAGCTTCGAATTCAATATGGCGCAGTCCAAGCACGGCCTGACCAAGGAGATGACCAT
A_WT-24	GCCCTCCATGCGGTACTTCATGGTCATCTCCTTGGTCAGGCCGTGCTTGGACTGGGCCATATTGAATTCGAAGCTTGAGCTCGAGATCTGAGTC
B_WT-52	TGATCACGAACTTGTGGCCGTCCACGCAGCCCTCCATGCGGTACTTCATGGTCATCTCCTTGGTCAGGCCGTGCTTGGACTGGGCCATATTGAATTCGAAGCTTGAGCTCGAGATCTGAGTC
C_WT-97	TGATGGCCTGCTTGCCCTTGAAGGGGTAGCCGATGCCCTCGCCGGTGATCACGAACTTGTGGCCGTCCACGCAGCCCTCCATGCGGTACTTCATGGTCATCTCCTTGGTCAGGCCGTGCTTGGACTGGGCCATATTGAATTCGAAGCTTGAGCTCGAGATCTGAGTC
A_mut_24	GCCCTCCATGCGGTACTTCATGGTCATCTCTTTGGTCAGGCCGTGCTTGGACTGGGCCATATTGAATTCGAAGCTTGAGCTCGAGATCTGAGTC
B_mut_52	TGATCACGAACTTGTGGCCGTCCACGCAGCCCTCCATTCGGTACTTCATGGTCATCTCCTTGGTCAGGCCGTGCTTGGACTGGGCCATATTGAATTCGAAGCTTGAGCTCGAGATCTGAGTC
C_mut_97	TGATGGCCTGCTTGCCCTTGAAGGGGTAGCCGATTCCCTCGCCGGTGATCACGAACTTGTGGCCGTCCACGCAGCCCTCCATGCGGTACTTCATGGTCATCTCCTTGGTCAGGCCGTGCTTGGACTGGGCCATATTGAATTCGAAGCTTGAGCTCGAGATCTGAGTC

*:7_optimized ssODN shown in Fig.5, **T/A**:target base (ACG→ATG), **Red**: blocking mutation,
underline: gRNA , double underline: PAM (w or w/o blocking mutation)

Table S3. Knock-in efficiency by sgRNAs inducing DSB at distal sites from target base for substitution

ssODN	sgRNA	Distance * (nt)	ssODN length			KI %	KI%/total Edited cell***
			total (nt)	HA-L**(nt)	HA-R**(nt)		
7_optimized ssODN	original	10	75	35	30	28.8±0.6	43.0±0.4
A_WT-24	A	24	94	35	35	1.0±0.3	1.1±0.3
B_WT-52	B	52	122	35	35	0.2±0.1	0.2±0.1
C_WT-97	C	97	167	35	35	0.1±0.1	0.1±0.1
A_mut_24	A	24	94	30	35	2.2±0.1	2.8±0.1
B_mut_52	B	52	122	35	35	1.1±0.2	1.6±0.3
C_mut_97	C	97	167	34	35	0.2±0.1	0.2±0.1

*: the distance from the DSB site to the target base.
**: length of the perfect homology arm at left and right ends.
***: KI % in all genome-edited cells (KI % + Indel %)
